# The Rheumatoid Arthritis Risk Variant CCR6DNP Regulates *CCR6* via PARP-1

**DOI:** 10.1371/journal.pgen.1006292

**Published:** 2016-09-14

**Authors:** Gang Li, Pierre Cunin, Di Wu, Dorothée Diogo, Yu Yang, Yukinori Okada, Robert M. Plenge, Peter A. Nigrovic

**Affiliations:** 1 Division of Rheumatology, Immunology and Allergy, Brigham and Women’s Hospital, Boston, Massachusetts, United States of America; 2 Department of Statistics, Harvard University, Cambridge, Massachusetts, United States of America; 3 Centre for Cancer Research, Monash Institute of Medical Research, Monash University, Clayton, Victoria, Australia; 4 Division of Genetics, Brigham and Women's Hospital, Harvard Medical School, Boston, Massachusetts, United States of America; 5 Program in Medical and Population Genetics, Broad Institute, Cambridge, Massachusetts, United States of America; 6 Department of Human Genetics and Disease Diversity, Graduate School of Medical and Dental Sciences, Tokyo Medical and Dental University, Tokyo, Japan; 7 Laboratory for Statistical Analysis, RIKEN Center for Integrative Medical Sciences, Yokohama, Japan; 8 Department of Statistical Genetics, Osaka University Graduate School of Medicine, Osaka, Japan; 9 Division of Immunology, Boston Children’s Hospital, Boston, Massachusetts, United States of America; University of Chicago Department of Medicine, UNITED STATES

## Abstract

Understanding the implications of genome-wide association studies (GWAS) for disease biology requires both identification of causal variants and definition of how these variants alter gene function. The non-coding triallelic dinucleotide polymorphism CCR6DNP is associated with risk for rheumatoid arthritis, and is considered likely causal because allelic variation correlates with expression of the chemokine receptor CCR6. Using transcription activator-like effector nuclease (TALEN) gene editing, we confirmed that CCR6DNP regulates *CCR6*. To identify the associated transcription factor, we applied a novel assay, Flanking Restriction Enhanced Pulldown (FREP), to identify specific association of poly (ADP-ribose) polymerase 1 (PARP-1) with CCR6DNP consistent with the established allelic risk hierarchy. Correspondingly, manipulation of PARP-1 expression or activity impaired CCR6 expression in several lineages. These findings show that CCR6DNP is a causal variant through which PARP-1 regulates *CCR6*, and introduce a highly efficient approach to interrogate non-coding genetic polymorphisms associated with human disease.

## Author Summary

Genome-wide association studies (GWAS) identify loci associated with human disease risk, but bridging the gap between locus and mechanism has proven particularly difficult in cases where associated variants do not alter coding. We aimed to develop a generalizable approach to this problem. Previously, a dual nucleotide polymorphism within the first intron of *CCR6* (termed the CCR6DNP) had been associated with risk for rheumatoid arthritis, but the pathway by which this variant altered gene expression could not be determined. Here, we employed sequence perturbation to confirm a regulatory role for the CCR6DNP. Next, using a new technique termed Flanking Restriction Enhanced Pulldown (FREP), we identified PARP-1 as the protein that regulates *CCR6* expression through allelic association with the CCR6DNP, a finding confirmed by chromatin immunoprecipitation and functional assays. These findings reveal an unexpected regulatory pathway for *CCR6* implicated in rheumatoid arthritis and other disease by human genetics, and more generally introduce a novel approach to identifying regulatory protein-DNA interactions.

## Introduction

Rheumatoid arthritis (RA) is an autoimmune disease that affects 0.5–1% of the population, resulting in destructive inflammation of the joints and other tissues. The pathogenesis of RA remains incompletely understood [1]. GWAS have identified over 100 associated loci, confirming remarkable genetic complexity [2]. For many of these loci, the responsible genetic polymorphism remains ambiguous, in particular for loci that are not in linkage dysequlibrium (LD) with any variant that affects protein sequence. This ambiguity complicates the optimal utilization of human genetics to understand disease pathogenesis and to identify new therapeutic targets [3].

For some loci, however, specific non-coding variants have been implicated in disease risk. One example is the RA risk locus at 6q37. While several genes reside in this locus, integrative bioinformatics approaches implicate *CCR6* as the likely risk gene [2],[4]. This suggestion is supported by biological plausibility. CCR6 is expressed by T cells, including Th17 and Treg subtypes, dendritic cells, and B cells, and plays a role in cell recruitment during inflammation [5]. CCL20, the only known ligand for CCR6, is produced within the inflamed joint by cells including fibroblast-like synoviocytes, neutrophils, and Th17 cells [5]. Murine studies confirm that CCR6 antagonism can attenuate experimental arthritis [6]. Thus, understanding how genetic variants around *CCR6* influence RA risk could shed new light on disease pathogenesis.

In 2010, Kochi, Okada et al. identified a novel triallelic dinucleotide polymorphism, CCR6DNP, as the likely non-coding variant regulating *CCR6* expression [4]. CCR6DNP alleles enhanced CCR6 expression in a luciferase reporter assay and correlated with greater expression of CCR6 in Epstein-Barr virus-transformed lymphoblastoid cell lines, in parallel with the order of RA risk (TG>CG>CA). RA patients carrying higher risk alleles were more likely to have detectable circulating levels of IL-17. Finally, binding of nuclear protein(s) in an allele-specific manner was observed using an electrophoretic mobility shift assay (EMSA). However, bioinformatic and candidate approaches were unsuccessful in defining a specific transcriptional regulator. These data provide strong, but still correlative, support for the hypothesis that CCR6DNP regulates *CCR6*.

Identification of the specific protein or protein complex that regulates *CCR6* through CCR6DNP would be important. First, it would confirm that CCR6DNP is indeed the direct regulatory variant. Second, it would define the cellular pathway that regulates *CCR6* expression and thereby open up the potential for therapeutic targeting, not only for RA but also for other inflammatory diseases associated with the CCR6DNP, including Crohn’s disease, Graves’ disease, and systemic sclerosis [4,7].

However, identification of specific regulatory proteins is difficult. Traditional DNA pulldown assays are complicated by extensive binding of non-specific DNA binding proteins. This technical limitation represents an important roadblock in the effort to “bridge the gap” from GWAS to mechanism for polymorphisms that do not alter protein coding.

Here, we sought to identify the mechanism by which the CCR6DNP regulates *CCR6*. We first confirmed its regulatory function through TALEN gene editing at the CCR6DNP locus. We then applied a novel DNA pulldown method, termed Flanking Restriction Enhanced Pulldown (FREP), that reduces non-specific binding to the DNA probe through sequential enzymatic restriction and that can further be employed in an allelic competition assay to test sequence-specific binding. Using FREP, we identified a specific allelic association between the CCR6DNP and PARP-1, a protein previously implicated in murine arthritis [8]. This result was confirmed by chromatin immunoprecipitation (ChIP) and genetic targeting as well as enzymatic inhibition of PARP-1. These findings begin to define the mechanism through which CCR6DNP regulates *CCR6* and more generally model an efficient approach to proceed from regulatory polymorphism to molecular mechanism.

## Results

### Mutations at the CCR6DNP locus alter CCR6 expression

**Fig 1:**
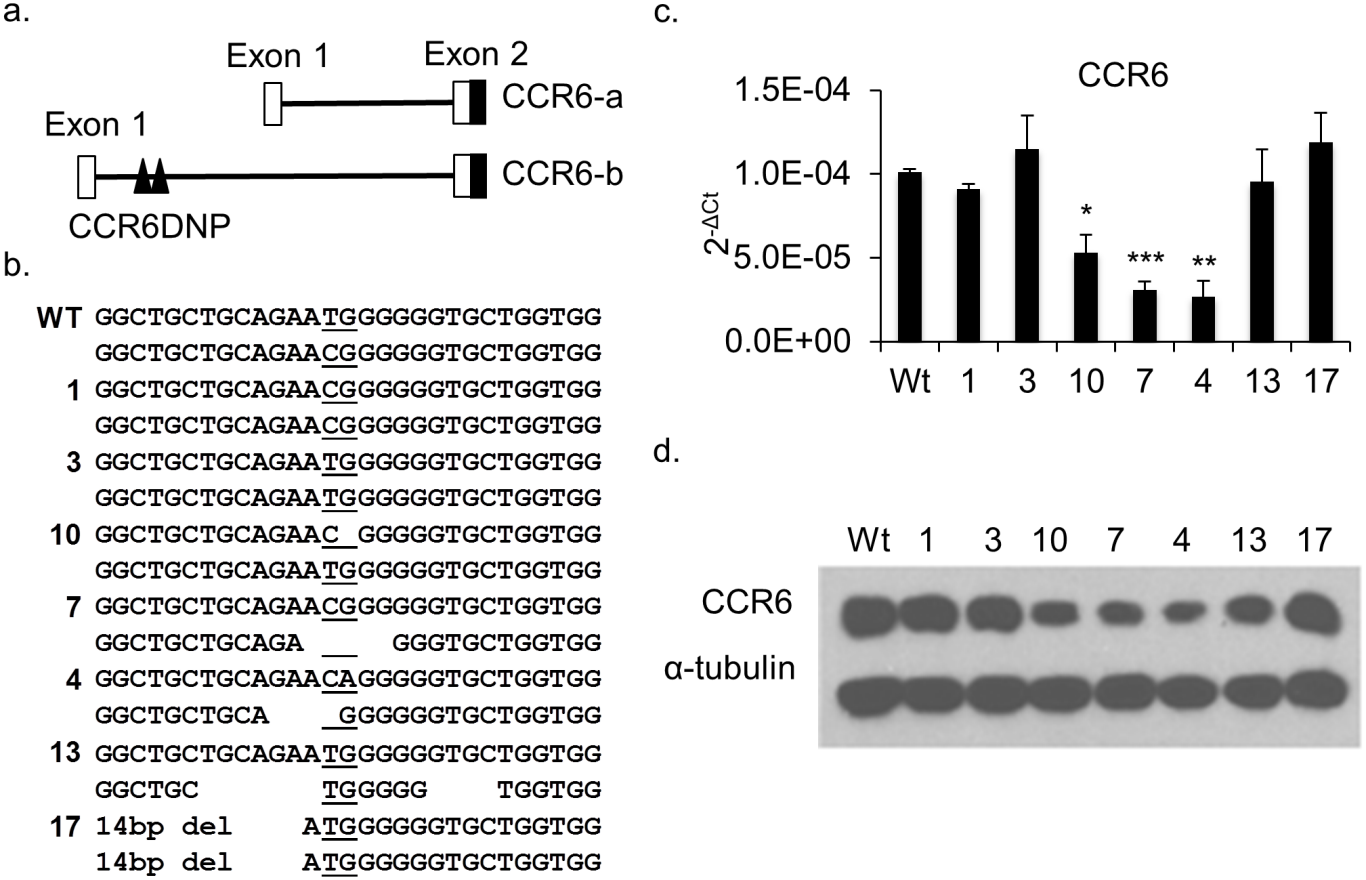
Characterization of mutated HCT116 clones generated by TALENs targeting the CCR6DNP. (a) Partial genomic arrangement of *CCR6* showing alternative transcripts CCR6-a and CCR6-b and the location of CCR6DNP. Only CCR-b could be detected in HCT116 and Jurkat T cells by real time PCR (see S3 Fig). (b) Sequence of the three alleles of CCR6DNP in 7 representative targeted HCT116 clones. The CCR6DNP is underlined. (c) and (d) Expression of CCR6 in the 7 targeted clones by qPCR and Western blot. For qPCR, data are shown as mean±s.d. (*n* = 3). Sequence, sequence trace and expression data for all 17 mutated HCT116 clones are in S1 and S2 Figs. Statistical significance in 1c reflects comparison to WT.

The CCR6DNP resides in intron 1 of *CCR6* (Fig 1a). To confirm the role of the CCR6DNP in gene expression we employed TALEN gene editing [9]. For these experiments we employed HCT116 cells, a human colon cancer line that expresses CCR6 at a high level and is easily transfectable. Cells were co-transfected with both left and right TALEN constructs together with a puromycin selection marker. After selection, we screened more than 100 puromycin-resistant clones by Sanger sequencing of a 122bp PCR fragment flanking the CCR6DNP. Clones positive for mutations at the CCR6DNP locus were sub-cloned. Identical 122bp PCR fragments from these sub-clones were cloned into TA vectors for sequencing to define the mutations on each allele. In total, we obtained 17 mutated clones, including 18 deletions and 4 mutated alleles (Fig 1b and S1 Fig). We then measured CCR6 expression by both qPCR and Western blot, confirming that in most clones sequence perturbation at CCR6DNP modulated gene expression (Fig 1c and 1d; S2a Fig). By contrast, expression of the control gene *CD40* was unchanged (S2b Fig). This result confirms that CCR6DNP participates directly in the regulation of *CCR6* and is thus a plausible causal variant at the RA-associated 6q27 locus.

### Flanking Restriction Enhanced Pulldown (FREP) identifies the association of PARP-1 with the CCR6DNP

**Fig 2:**
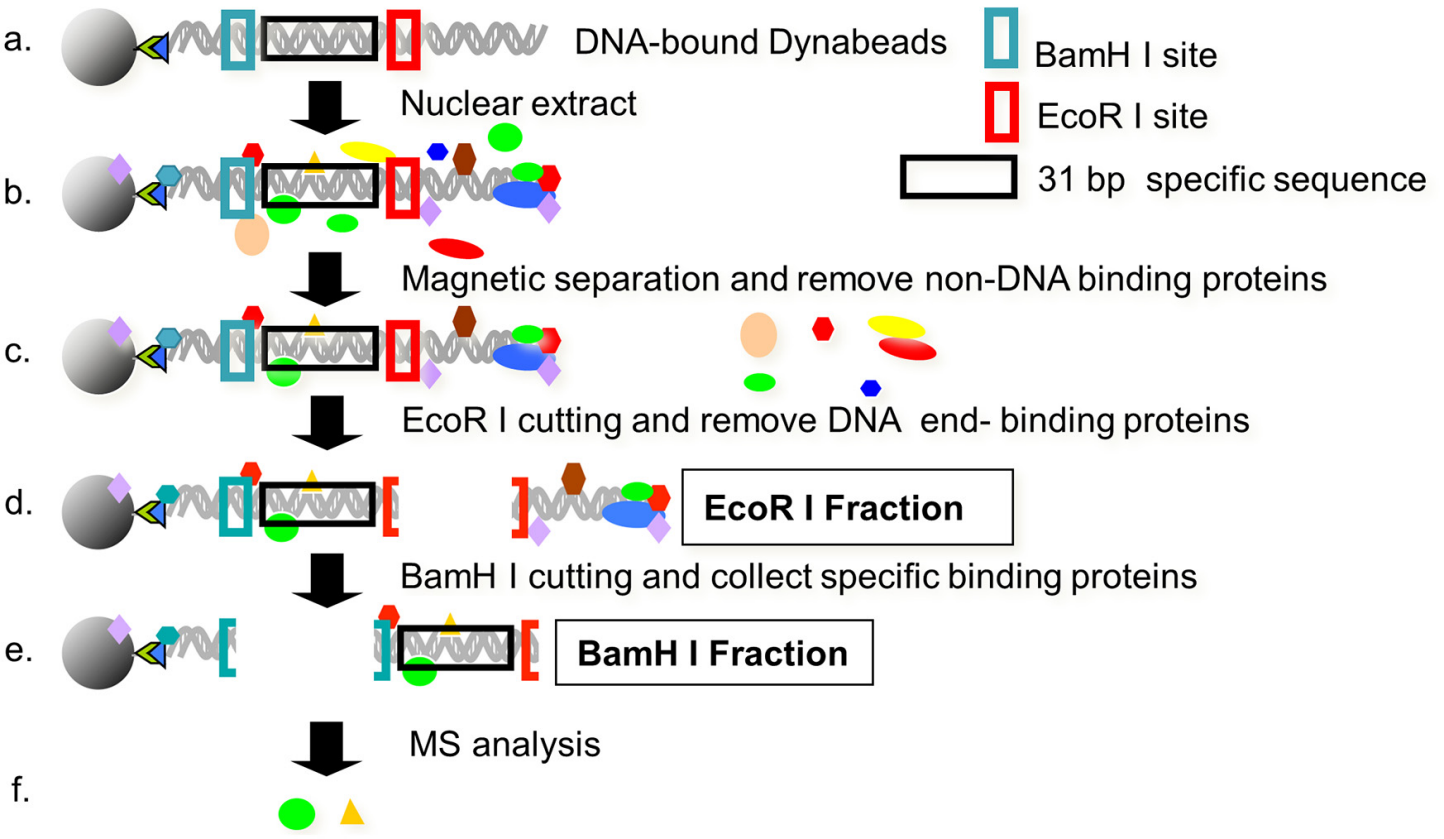
Flanking Restriction Enhanced Pulldown (FREP). (a) An 82 bp biotinylated DNA fragment is conjugated to streptavidin-coated magnetic Dynabeads (Invitrogen). This fragment is engineered to include a 31bp gene specific (“bait”) sequence (black box), flanked by restriction enzyme cleavage sites for BamH I proximally (blue box) and EcoR I distally (red box), as well as 20bp DNA fragments to allow PCR amplification of the whole unit. (b) DNA-beads are mixed with nuclear extract. A free 82 bp non-biotinylated DNA fragment can be included in the control reaction at this stage as a specific competitor. (c) Magnetic separation and wash to remove non-DNA binding proteins. (d) EcoR I digestion to release 3’ DNA end-binding proteins, yielding the EcoR I fraction. (e) BamH I digestion to separate the sequence specific DNA binding proteins, BamH I fraction, from proteins that bind 5’ DNA and Dynabeads. (f) Mass spectrometry (MS) to identify proteins remaining within the BamH I fraction.

**Fig 3:**
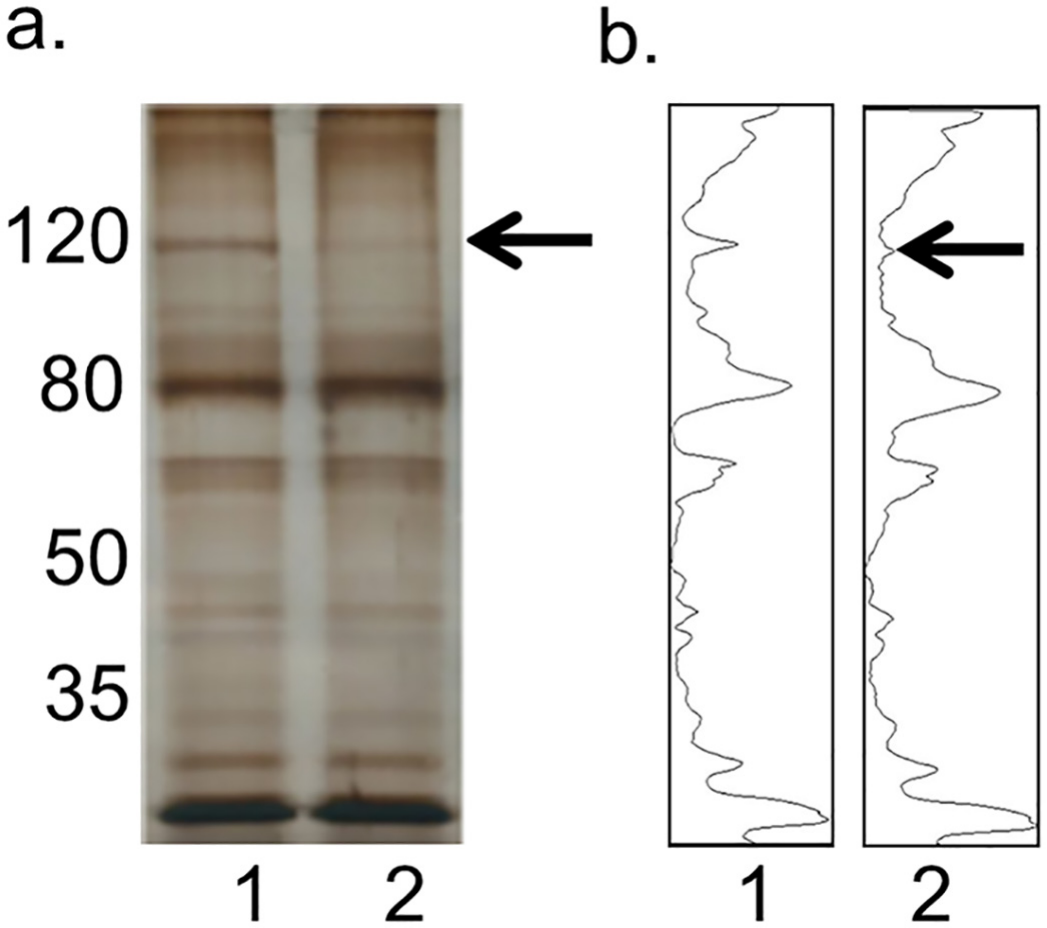
FREP to identify the binding of PARP-1 with CCR6DNP. (a) Silver stain and (b) densitometry to show proteins pulled down from CCR6DNP/TG-beads mixed with nuclear extract from THP-1 cells. Lane 1, BamH I fraction; Lane 2, BamH I fraction where pulldown was performed in the presence of a 40x excess of competitor (free CCR6DNP/TG). Arrow indicates the specific band sent for mass spectrometry analysis.

We assumed that CCR6DNP, as a causal allele located in an intron, required a transcription factor or enhancer to carry out its regulatory function. To identify such a protein, we developed a novel DNA pulldown assay termed FREP (Fig 2). An 82bp biotinylated DNA fragment was conjugated to streptavidin-coated magnetic Dynabeads (Invitrogen). This fragment was engineered to include a 31bp sequence containing the CCD6DNP (TG allele, termed CCR6DNP/TG), flanked by restriction enzyme cleavage sites for BamH I proximally and EcoR I distally, as well as 20bp sequences to allow PCR amplification of the whole unit. This configuration allows pulldown of DNA-binding proteins specific to the sequence of interest and also elimination of proteins that bind non-specifically to DNA. CCR6DNP/TG beads were incubated with nuclear extract from THP-1 cells, a human monocyte leukemia line expressing of CCR6. Following magnetic separation and wash, the CCR6DNP/TG beads were cut with EcoR I and the supernatant was collected as the EcoR I fraction. After a second round of magnetic separation and wash, the DNA beads were cut with BamH I to release the CCR6DNP/TG fragment together with any binding proteins. In parallel, we performed a control FREP in the presence of a 40-fold excess of free CCR6DNP/TG as a binding competitor. BamH I fractions from these experiments were resolved by SDS-PAGE and silver stained to identify potential CCR6DNP/TG binding proteins (Fig 3). A dominant band emerged at approximately 120kD (lane 1, indicated by an arrow) but was much lower in intensity in the presence of competitor (lane 2). This band was cut and analyzed by mass spectrometry, revealing 27 peptide fragments from a single protein, PARP-1 (Table 1).

**Table 1:** Mass spectrometry analysis identifies PARP-1 as the CCR6DNP binding protein.

Identified protein	Molecular weight	# peptide in blank control	# peptide in sample
PARP1_HUMAN	113 kDa	0	27
ALBU_BOVIN	69 kDa	1	16
K2C1_HUMAN (+1)	66 kDa	13	2
K1C9_HUMAN	62 kDa	10	2
K1C10_HUMAN	59 kDa	10	0
K22E_HUMAN	65 kDa	8	0

### The PARP-1 association with CCR6DNP exhibits sequence and allele specificity

**Fig 4:**
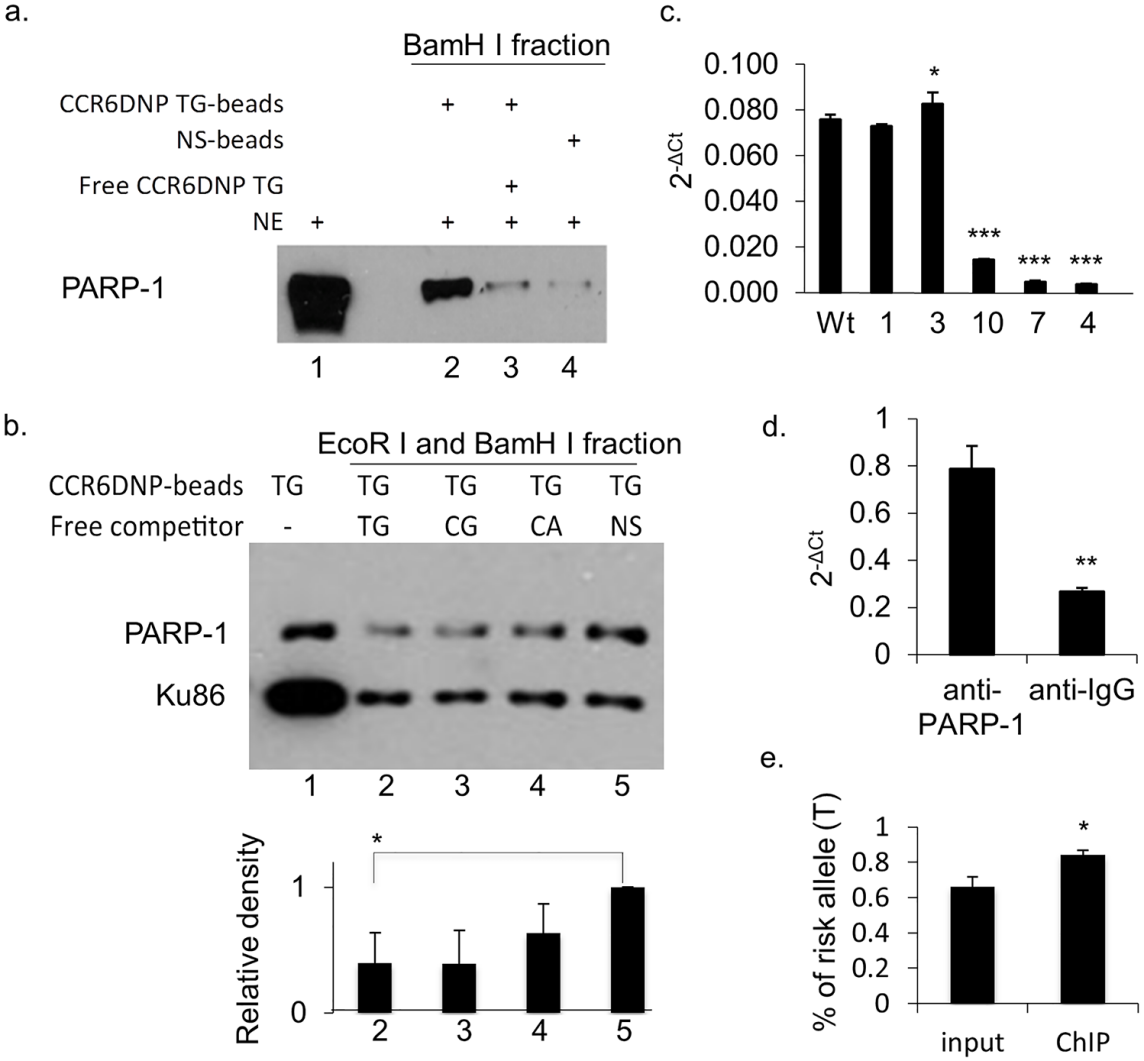
The association of PARP-1 with CCR6DNP is sequence dependent and allele specific. (a) FREP+Western blot for PARP-1 with BamH I fractions. Lane 1: nuclear extract (NE) input, lane 2: NE with CCR6DNP/TG-beads, lane 3: NE with CCR6DNP/TG-beads with cold competitor (Free TG), and lane 4: NE with a non-specific DNA sequence (NS-beads). (b) To detect allele specificity, FREP was modified to cut directly with BamH I only, yielding a combined BamH I+EcoR I fraction, and SDS-PAGE was probed with anti-PARP-1. Anti-Ku86 antibody was used as the internal loading control to ensure comparable amounts of competitor DNA. CCR6DNP/TG-beads were incubated with NE and competed with equal amount of free competitors. Lane 1: no competitor; lane 2: free CCR6DNP/TG; lane 3: CCR6DNP/CG; lane 4: free CCR6DNP/CA; and lane 5: free non-specific DNA. Upper: Western blot; Lower: relative density from the Western blot. (c) ChIP assay with an anti-PARP-1 antibody on WT HCT116 cells and 5 mutants as indicated in Fig 1 showing impaired binding of PARP-1 to the CCR6DNP. (d) ChIP assay with an anti-PARP-1 antibody on WT Jurkat T cells showing significant enrichment of the CCR6DNP fragments comparing to anti-IgG antibody. (e) Sequencing of the CCR6DNP fragments from the ChIP with WT HCT116 cells showing the significant enrichment of the T allele over the C allele by an anti-PARP-1 antibody. Results from (a), (b), (c), and (d) representative of 3 experiments. Data are shown as mean±s.d. (*n* = 3).

PARP-1 is a well-known DNA repair protein [10]. It is a DNA end-binding protein and can also bind single-stranded DNA without sequence specificity [11]. However, FREP minimizes detection of such non-specific binding through restriction enzyme digestion, because the 3’ free end is cleaved off by EcoR I, while single-stranded DNA is not cleaved by BamH I and is therefore discarded along with the bead. To confirm sequence-specific association of PARP-1 with CCR6DNP/TG, we combined FREP with Western blot, adapting a method developed by Wu [12]. We compared FREP using CCR6DNP/TG with FREP using a non-specific 31bp DNA sequence, and probed the Western blot of BamH I fractions with an anti-PARP-1 antibody (Fig 4a). Our results showed that PARP-1 associated much more strongly with CCR6DNP/TG (lane 2) than with the non-specific DNA sequence (lane 4). This association could be specifically competed away by the free CCR6DNP/TG (lane 3). In this assay, some PARP-1 was also observed with the non-specific DNA sequence, likely reflecting end binding to DNA uncut by EcoR I. Together, these results demonstrate that PARP-1 binds CCR6DNP/TG with sequence specificity.

We then investigated whether PARP-1 exhibited allele-specific association with CCR6DNP in accordance with the order of RA risk TG>CG>CA [4]. For this purpose, we repeated FREP using CCR6DNP/TG beads in the presence of excess free competitors CCR6DNP/TG, CCR6DNP/CG, CCR6DNP/CA, or a non-specific DNA sequence (Fig 4b; lane 2, 3, 4 and 5, respectively). In these experiments, CCR6DNP/TG-beads were mixed with nuclear extract and different competitors, after which the beads were washed and separated. The bead-bound CCR6DNP/TG was cut directly with BamH I and the supernatant was resolved on SDS-PAGE. Western blots were probed with an anti-PARP-1 antibody. In triplicate experiments, the association of PARP-1 with CCR6DNP/TG was competed away by free CCR6DNP/TG more efficiently than by non-specific DNA (p<0.05, n = 3), with a trend toward TG>GC>CA in line with the established CCR6DNP risk hierarchy (Fig 4b; lane 2, 3, and 4, respectively). Non-specific DNA also competed for PARP-1 binding, but less efficiently, consistent with the known end-binding activity of PARP-1 (Fig 4b; lane 5) [11,13]. To ensure equivalent concentrations of competitors, we probed simultaneously against Ku86, a double-stranded DNA end-binding protein without known sequence specificity. All four competitors inhibited Ku86 binding equally. Together, these data show that PARP-1 associates with the CCR6DNP in an allelic manner consistent with the RA risk hierarchy TG>CG>CA.

To further confirm the association of PARP-1 with CCR6DNP, we performed ChIP assays using an anti-PARP-1 antibody (Genetex GTX100573). First, we performed ChIP using wild type HCT116 cells and the mutated HCT116 clones #1, 3, 4, 7 and 10 as shown in Fig 1. In brief, cells from different clones were cross-linked using 1% formaldehyde, fragmented with sonication, and followed by immunoprecipitation. After the cross-linked DNAs were reversed and purified, the endogenous association of PARP-1 with CCR6DNP was detected by qPCR using primers recognizing a shared sequence 5’ of the CCR6DNP and outside of the mutated regions. Our data showed that the binding of PARP-1 to CCR6DNP corresponds with the levels of CCR6 expression in these mutants in Fig 1 (Fig 4c). Second, we performed ChIP with human Jurkat T cells, confirming that the association of PARP-1 with CCR6DNP is evident not only in HCT116 but also in T cells (Fig 4d). Further, we sequenced the CCR6DNP fragments from the ChIP with WT HCT116 cells in Fig 4c, which as shown in Fig 1b are heterozygous TG/CG at the CCR6DNP. CCR6DNP fragments were cloned by PCR into TA vector. Random sequencing of both alleles revealed significant enrichment of the risk allele T, consistent with preferential binding of PARP-1 to the TG allele (Fig 4e). Together, these results support the conclusion that PARP-1 associates with the CCR6DNP in an allele-specific manner.

### PARP-1 regulates CCR6 expression in human HCT116 cells and Jurkat T cells

**Fig 5:**
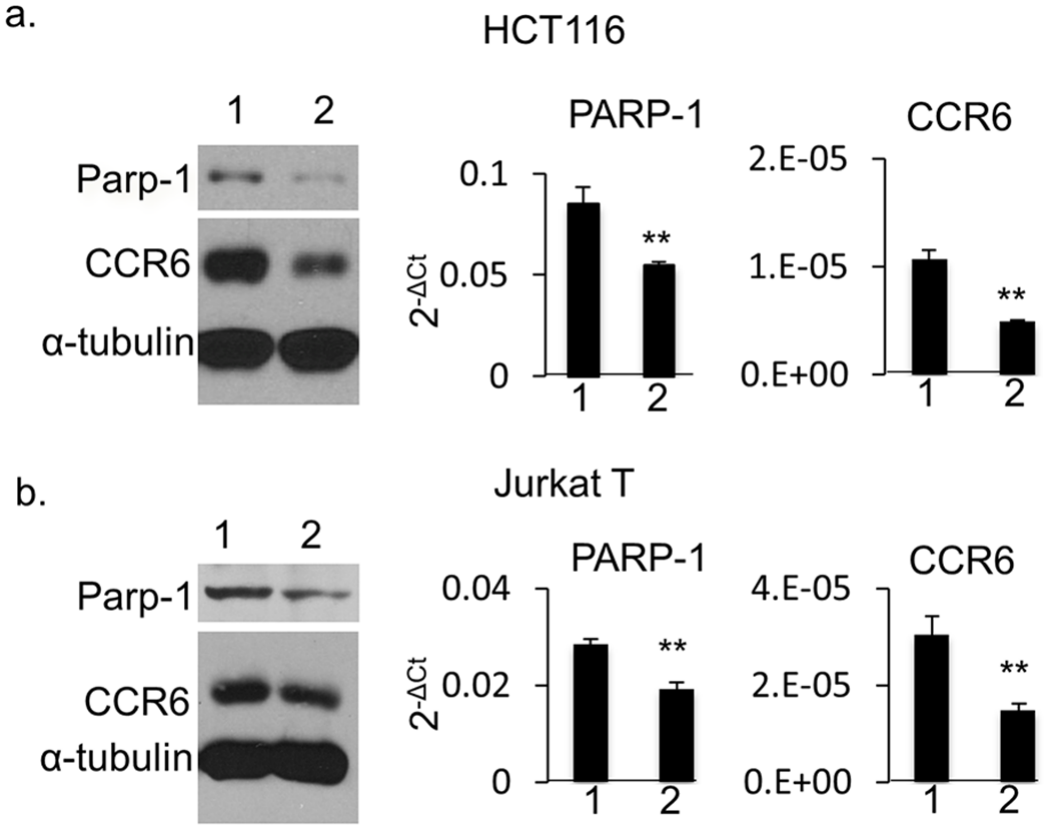
CCR6 expression in PARP-1 knockdown human cells. HCT116 cells (a) and Jurkat T cells (b) by Western blot (left), and qPCR analysis on PARP-1 (middle) and on CCR6 (right). For Western blot, whole cell extract was isolated and separated on SDS-PAGE gel. Western blot was detected with mouse anti-human PARP-1, CCR6 and α-tubulin antibodies simultaneously. Lane 1: negative control for shRNA. Lane 2: shRNA treatment for HCT116 cells or siRNA treatment for Jurkat T cells. For qPCR, data are shown as mean±s.d. (*n* = 3).

The association of PARP-1 with CCR6DNP suggests that PARP-1 is a regulatory protein that regulates *CCR6*. To test this hypothesis, we generated PARP-1-deficient HCT116 cells by shRNA knockdown. PARP-1-specific shRNA, but not scrambled shRNA, induced a striking reduction of CCR6 expression by both Western blot (Fig 5a, left) and qPCR analysis (Fig 5a, middle and right). To ensure that this mechanism is not restricted to HCT116 cells, we performed siRNA knockdown of PARP-1 in human Jurkat T cells and confirmed a similar result (Fig 5b). These results demonstrate that PARP-1 is a novel regulator of CCR6 expression across human cell types.

### The (ADP-ribose) polymerase activity of PARP-1 participates in the regulation of *CCR6* in human cells

**Fig 6:**
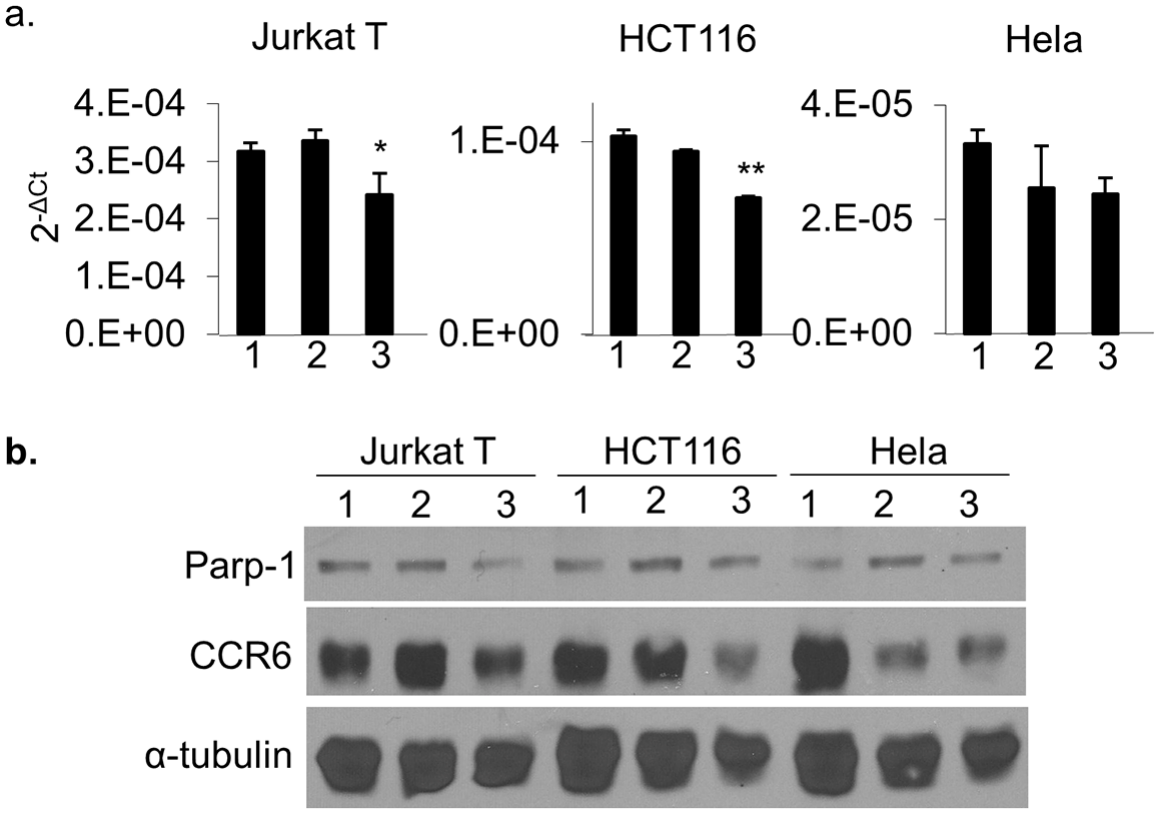
CCR6 expression in human cells treated with 3-aminobenzamide. (a) qPCR and (b) Western blot to show expression of CCR6 and PARP-1 in Jurkat T cells, HCT116 and Hela cells. Cells were treated with 0 mM (lane 1), 5 mM (lane 2) and 10 mM (lane 3) 3-AB for 72 hrs. For qPCR, data are shown as mean±s.d. (*n* = 3).

PARP-1 is a poly(ADP-ribose)polymerase that transfers ADP-ribose groups to target proteins and can thereby alter gene transcription [10]. To test if poly(ADP-ribose)polymerase activity is required for the regulation of CCR6 expression, we treated human Jurkat T cells, HCT116 cells and Hela cells with a PARP-1 inhibitor, 3-aminobenzamide (3-AB), that inhibits PARP-1 enzymatic activity by competing for the NAD+ binding site on PARP-1 [14]. Cells were treated with 0, 5 and 10 mM 3-AB for 72 h and analyzed for CCR6 expression by qPCR and Western blot (Fig 6a and 6b). At the 10 mM concentration, our results showed a significant decrease of CCR6 expression on Jurkat T cells and HCT116, while Hela cells exhibited a trend in the same direction. Under these conditions, treated cells grew at a slower rate (~75% of control), but no apoptosis was observed morphologically or by Western blot, as PARP-1 degradation is a marker of apoptosis (Fig 6b). This result confirms the identification of PARP-1 in the regulation of CCR6 expression and implicates its poly(ADP-ribose)polymerase activity in this function.

## Discussion

GWAS have identified a role for numerous genetic loci in the modulation of complex phenotypes, including common diseases such as RA. Dissecting the associated mechanistic pathways remains a major challenge, especially for non-coding variants, even when a putative causative polymorphism has been established [3]. In this report, we introduce FREP as a technically straightforward tool to identify DNA binding proteins, and use this method to define the pathway by which a functional polymorphism, the CCR6DNP, regulates expression of *CCR6*. Specifically, we show that PARP-1 associates with the CCR6DNP in sequence- and allele-specific manner; that interference with PARP-1 expression impairs expression of CCR6 at mRNA and protein levels; and that chemical inhibition of PARP-1 exerts a similar effect, indicating that the enzymatic activity of PARP-1 is relevant for its regulatory function, while confirming that the pathway spans cellular lineages.

Compared with conventional DNA pulldown assays [15], FREP reduces non-specific binding through distinct restriction enzyme cleavage sites on either side of the bait sequence. An identical DNA construct, but without bead linkage, can be employed as a competitor to highlight proteins that bind bait DNA with sequence specificity (see Fig 3). We further adapted a method developed by Wu [12] to combine DNA pulldown with Western blot, in the setting of varying competitor sequences, to assess allelic specificity (see Fig 4b). Together, these techniques provide a new approach to explore regulatory DNA-protein associations identified by genetic association studies.

FREP and other pulldown assays cannot distinguish direct DNA binding from participation in a larger DNA-binding protein complex. Previous reports have implicated PARP-1 in transcriptional regulation of a number of human genes including *HFE*, *CXCL1*, *cTnT*, *iNOS*, *SNCA* and *Tcirg1* [16–24]. In these reports, PARP-1 interacted with the TGTTG sequence in the *cTnT* promoter, GCTGTGGGAA in the *Tcirg1* promoter, and the palindrome-like sequence ATGGTcttACCTA in the *HFE* promoter. The lack of sequence specificity could suggest that PARP-1 serves as part of a larger protein complex, or alternately that it recognizes a secondary structure such as cruciform DNA [25,26]. Indeed, our present data do not establish that PARP-1 itself directly recognizes the CCR6DNP sequence. However, it is interesting to note that the CCR6DNP risk allele GCAGAA**TG**GGGGGT bears some similarity to the binding sequences at *cTnT* and *Tcirg1*, suggesting that—if in fact PARP-1 binds directly to these sequences—then TG might play an important role in DNA recognition.

PARP-1 is the founding member of a family of enzymes engaged in transfer of poly ADP-ribose (PAR) to a wide range of nuclear and cytoplasmic proteins, including PARP-1 itself [10,27]. PARP-1 is a multifunctional protein that plays essential roles in the cell, including DNA repair, translation, transcription, telomere maintenance, genomic stability, and chromatin remodeling [28]. PARP-1-deficient mice are viable but show increased sensitivity to genotoxic stress, resistance to DNA damage-induced cell death, and increased susceptibility to chemically- or genetically-induced tumors [29]. Recently, accumulating data suggest that PARP-1 is an important regulator of gene transcription [28–30]. This role may be accomplished in multiple ways, including binding to DNA, decondensation of chromatin [31,32], alteration of mRNA splicing through regulation of the splicing silencers or enhancers [33], recruitment of transcription factors [34], and post-translational modulation of RNA translation [35,36]. Some of these studies implicate the enzymatic activity of PARP-1 [24, 30], although in other contexts this activity appears dispensable [37]. Our findings add to this literature by identifying *CCR6* as a specific target of PARP-1, defining a disease-associated DNA polymorphism that modulates this association, and confirming a role for PARP-1 enzymatic activity in the transcriptional regulation of *CCR6*. Our data do not define the relevant PARylation target, which could include another protein in the *CCR6* regulatory complex, associated histones, or PARP-1 itself.

Identification of the role for PARP-1 as a regulator of *CCR6* began with experimental confirmation by TALEN that CCR6DNP was directly involved in modulation of gene expression, as had been suggested previously [4]. These experiments targeted the general locus, rather than the dinuclotide motif itself, and revealed clone-to-clone variability that we presume reflects the effect of each mutation on the larger binding sequence, not defined here. For example, deletions flanking the CCR6DNP in clone 9 translated into impaired *CCR6* expression despite an intact CCR6DNP (S1 and S2 Figs), while gene expression was hardly altered by mutations introduced in clones 13 and 17 (Fig 1). Despite this variability, these results helped to engender confidence that CCR6DNP was indeed a suitable “bait” region, a conclusion borne out by the subsequent identification of the allelic association of PARP-1 with this locus.

One strength of FREP is its utility to test allele specificity through a unique assay in which FREP is performed in the presence of allelic competitors (Fig 4b). This assay can be technically challenging if allelic impact on binding affinity is partial. For example, in triplicate repeats quantitated using densitometry, we were able to distinguish statistically between the high-risk TG allele and non-specific DNA, but found only a trend with respect to intermediate risk alleles GC and CA, though it is notable that no such trend was evident in the binding of loading control Ku86.

The effect of PARP-1 on *CCR6* is consistent with its role as an activator of inflammation mediated by NFκB, AP-1, and MAP kinases [38–40]. Its impact on *CCR6* via a RA-associated functional polymorphism is also consistent with the role of PARP-1 in inflammatory arthritis [8], although limited knowledge of signal transduction pathways downstream of CCR6 constrains the understanding of how the interaction between PARP-1 and CCR6 contributes to RA. Nevertheless, both genetic deficiency [41] and chemical inhibition [42–44] of PARP-1 attenuates experimental arthritis in the mouse. The latter is accompanied by significant reductions in pro-inflammatory cytokines, chemokines, inflammatory mediators, and endothelial expression of key adhesion molecules VCAM-1 and ICAM-1. Our finding that PARP-1 regulates *CCR6* –work originating in human genetics—lends further support to the hypothesis that PARP-1 could represent a promising anti-inflammatory target in humans, a conclusion of potential importance since PARP-1 inhibitors are already approved for human use in ovarian cancer [8,45].

More generally, our studies demonstrate a solution to the challenge of bridging the gap from GWAS to biological mechanism for regulatory polymorphisms, considered to represent a substantial fraction of GWAS hits [2,3]. First, TALEN or other targeted gene editing technique (such as clustered regularly-interspaced short palindromic repeats, CRISPR) is used to confirm that a specific locus is active in regulating a target gene. Second, FREP is employed to identify associated transcriptional enhancers or repressors. This approach is applicable both to the identification of regulatory proteins, as demonstrated here, and also to the validation of candidate regulatory proteins implicated through bioinformatic or other approaches.

In conclusion, we confirm that CCR6DNP is a causal allele that controls expression of *CCR6*, and identify PARP-1 as a direct regulator of *CCR6* through the RA risk polymorphism CCR6DNP. Our strategy, encompassing sequence perturbation and FREP, represents a novel approach to defining pathways by which non-coding genetic polymorphisms alter disease risk. A comprehensive understanding of such pathways will represent an important step forward in the development of individualized therapeutic strategies for patients with RA and other diseases.

## Materials and Methods

### Cells and culture

The human colon cancer line HCT116 and the human monocyte line THP-1 were purchased from ATCC and cultured in McCoy’s 5A and RPMI medium supplemented with 10% FBS, respectively. Mouse anti-human PARP-1 antibody (Cat#: 14-6666-92) and CCR6 antibody (Cat#: 14-1969-82) were purchased from eBioscience and used at 1μg/ml.

### Construction of TALEN expression plasmids

TALEN expression plasmids were constructed exactly as described [9]. The TALEN cloning backbone and TALE monomer template were purchased from Addgene (TALEN Kit #1000000019). All primers were synthesized and purchased from IDT. The TALEN target sequence for CCR6 is AGCACCCCCCATTCTGCAGC. Sequences for the left and right TALENs are TCATTCATGTTAGATCCACC and AGCCACAGCCCCCAGGGTGA.

### Generation of mutations by TALENs in HCT116 cells

50μg of right and left TALENs were transfected together with 5μg of pGK/puro (Addgene, plasmid 11349) using standard calcium phosphate transfection. After 48 h incubation, cells were re-plated and selected against 0.8 μg/ml puromycin. Single puromycin- resistant clones were harvested and genomic DNA isolated to amplify the CCR6 fragment using forward primer 5’-ACATGTCTCCCAAACTTCTCACCC-3’ and reverse primer: 5’-TTTGTGCAGGGAGGTTGGGATGAA-3’. The PCR fragments were either sequenced directly with the reverse primer or cloned into TA vector and sequenced with T7 primer (Genewiz). Clones positive for mutations were subcloned and their DNA was cloned into TA vector to sequence both alleles. Each clone was sequenced at least 10 times to establish heterozygosity or homozygosity.

### RNA isolation, reverse transcription and real time PCR

RNA was isolated using the RNeasy Mini Kit (Qiagen). cDNA was synthesized with SuperScript III First-Strand Synthesis System and cDNA was amplified by real time PCR with Power SYBR Green PCR Master Mix (Life Technologies). For detection of whole CCR6 mRNA (both a and b variants), 5’ primer: TGAGCGGGGAATCAATGAATT and 3’ primer: TCCTGCAAGGAGCACAGTAACAT were used. For detection of the transcript variant a, 5’ primer: TAGGAAGTGGCAATCCAGAAC and 3’ primer: ATGACTCCAGCTCACCAATG were used. For detection of the transcript variant b, 5’ primer: CCACGTGTATATGCTGGTGAA and 3’ primer: GGAGCTGTCTGTTCCACAAA were used.

### Flanking Restriction Enhanced Pulldown (FREP)

An 82bp DNA fragment biotinylated at the 5’ end by IDT was conjugated to streptavidin-coated Dynabeads as per manufacturer’s instructions (Invitrogen). This fragment was engineered to include a 31bp target sequence (see below) flanked by restriction enzyme cleavage sites with BamH I proximally and EcoR I distally. Outside these two cleavage sites were introduced 20bp DNA fragments for PCR amplification of the whole unit (proximal: 5’- AATGATACGGCGACCACCGA-3’; distal: 5’- CAAGCAGAAGACGGCATACGA-3’). Bead-linked DNA was mixed with 50μg THP-1 nuclear extract in 1x binding buffer (10 mM Tris pH 7.5, 50 mM KCl, 5mM MgCl2, 2.5% glycerol, 0.5% NP-40 and 1 μg polydI-dC) at room temperature for 20 min. Nuclear extract was generated by NE-PER nuclear and cytoplasmic extraction reagents (Thermo Scientific). After magnetic selection and wash with PBS+0.05% Tween 20, DNA beads with bound proteins were digested with EcoR I at 37C for 30 min. The supernatant was collected as the EcoR I fraction. After another magnetic selection and wash, the DNA beads were digested with BamH I at 37C for 30 min and the supernatant was collected as the BamH I fraction. Both EcoR I and BamH I fractions were resolved by 7% SDS-PAGE gel for either Western blot analysis or silver staining. Mass spectrometry was performed in BIDMC using Thermohybrid Orbitrap XL high resolution MS (Thermo Scientific).

The “bait” DNA fragment used for FREP was:

CCR6DNP/TG:

/5Biosg/AATGATACGGCGACCACCGAGGATCCGTGGCTGCTGCAGAA**TG**GGGGGTGCTGGTGAATTCTCGTATGCCGTCTTCTGCTTG.

The DNA fragments used for the competition assay were:

CCR6DNP/TG:

AATGATACGGCGACCACCGAGGATCCGTGGCTGCTGCAGAA**TG**GGGGGTGCTGGTGAATTCTCGTATGCCGTCTTCTGCTTG

CCR6DNP/CG:

AATGATACGGCGACCACCGAGGATCCGTGGCTGCTGCAGAA**CG**GGGGGTGCTGGTGAATTCTCGTATGCCGTCTTCTGCTTG

CCR6DNP/CA:

AATGATACGGCGACCACCGAGGATCCGTGGCTGCTGCAGAA**CA**GGGGGTGCTGGTGAATTCTCGTATGCCGTCTTCTGCTTG

Irrelevant DNA sequence: AATGATACGGCGACCACCGAGGATCCAGGGCTGTAGATTCCGGCCTGAAGCCTGGGAATTCTCGTATGCCGTCTTCTGCTTG.

### ChIP assay

The ChiP assay was performed as described in Noss et al., 2015 [46]. PARP-1 antibody was purchased from Genetex (GTX100573). The CCR6DNP fragment was detected by qPCR with 5’ primer: GTGAGAAGTTTGGGAGACATGT and 3’ primer TTGGAAACGCTCTAATAGACCAC. For assessing the frequency of both alleles on the CCR6DNP in wild type HCT116 cells before and after the ChIP assays, CCR6DNP fragments were cloned into TA vectors (Promega) and sequenced by T7 primer.

### PARP-1 knockdown

PARP-1 knockdown in HCT116 cells was performed using a mixture of four retroviruses containing 4 PARP-1 unique 29mer shRNAs, as per manufacturer’s instructions (Origene Cat#: TG315488). 24 h after infection, cells were re-plated and selected in 1 ug/ml puromycin. 3 days after selection, both protein and RNA were isolated for assays. PARP-1 knockdown in Jurkat cells was performed using PARP-1 siRNA from Santa Cruz Biotech (sc-29437). Transfection was done using GenMute siRNA Transfection Reagent for Jurkat Cell (SignaGen Laboratories) in a 24 well format. 40 pmoles siRNA was used. 48 h after transfection, protein and RNA were isolated for assay.

### 3-Aminobenzamide treatment

1x10^5^ cells were plated in a 24-well plate the day before the treatment. On day one, both DMSO (control) and different concentrations of 3-AB (Sigma) were added. After 72 hrs, protein and RNA were isolated for Western blot and qPCR assays.

### Statistical analysis

Statistical significance was evaluated with Student’s *t*-test, 2 tailed, *P<0.05, **P<0.01, ***P<0.001, NS, nonsignificant. *n* stands for the number of replicated independent experiments.

## Supporting Information

S1 Fig Sequence and sequence trace of 17 mutants generated by TALENs. (TIF)

S2 Fig Expression of CCR6 (a) and CD40 (b) in mutated HCT116 cells by real time PCR. (TIF)

S3 Fig Expression of CCR6 variants a and b in HCT116 and Jurkat T cells. (TIF)
