# Supplementary figures and images for: The Rheumatoid Arthritis Risk Variant CCR6DNP Regulates CCR6 via PARP-1
Source: PLoS Genet. 2016 Sep 14;12(9):e1006292. doi: 10.1371/journal.pgen.1006292 (PMC5023119; doi:10.1371/journal.pgen.1006292)

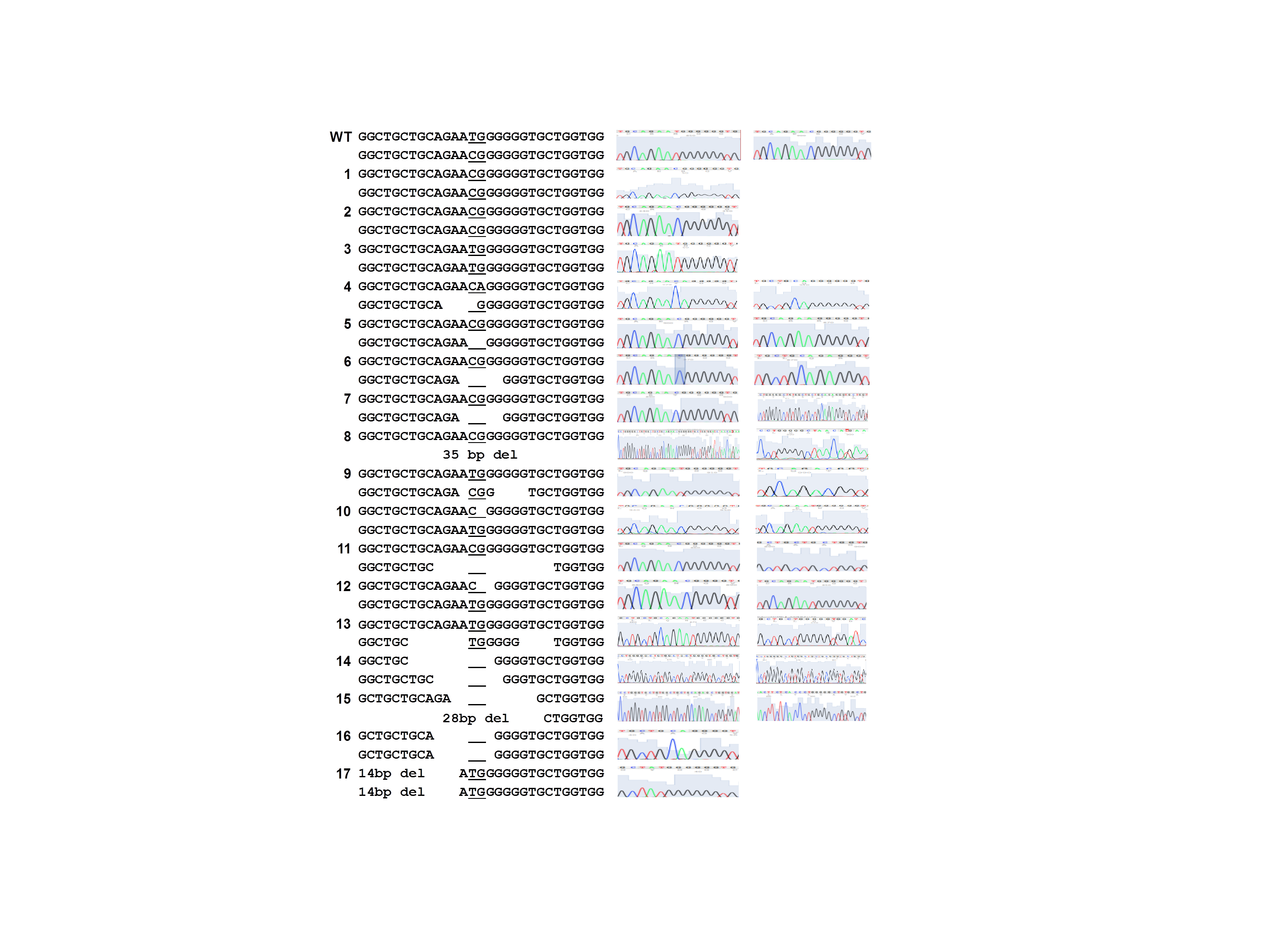

Supplement: S1 Fig — (TIF) [file pgen.1006292.s001.tif]

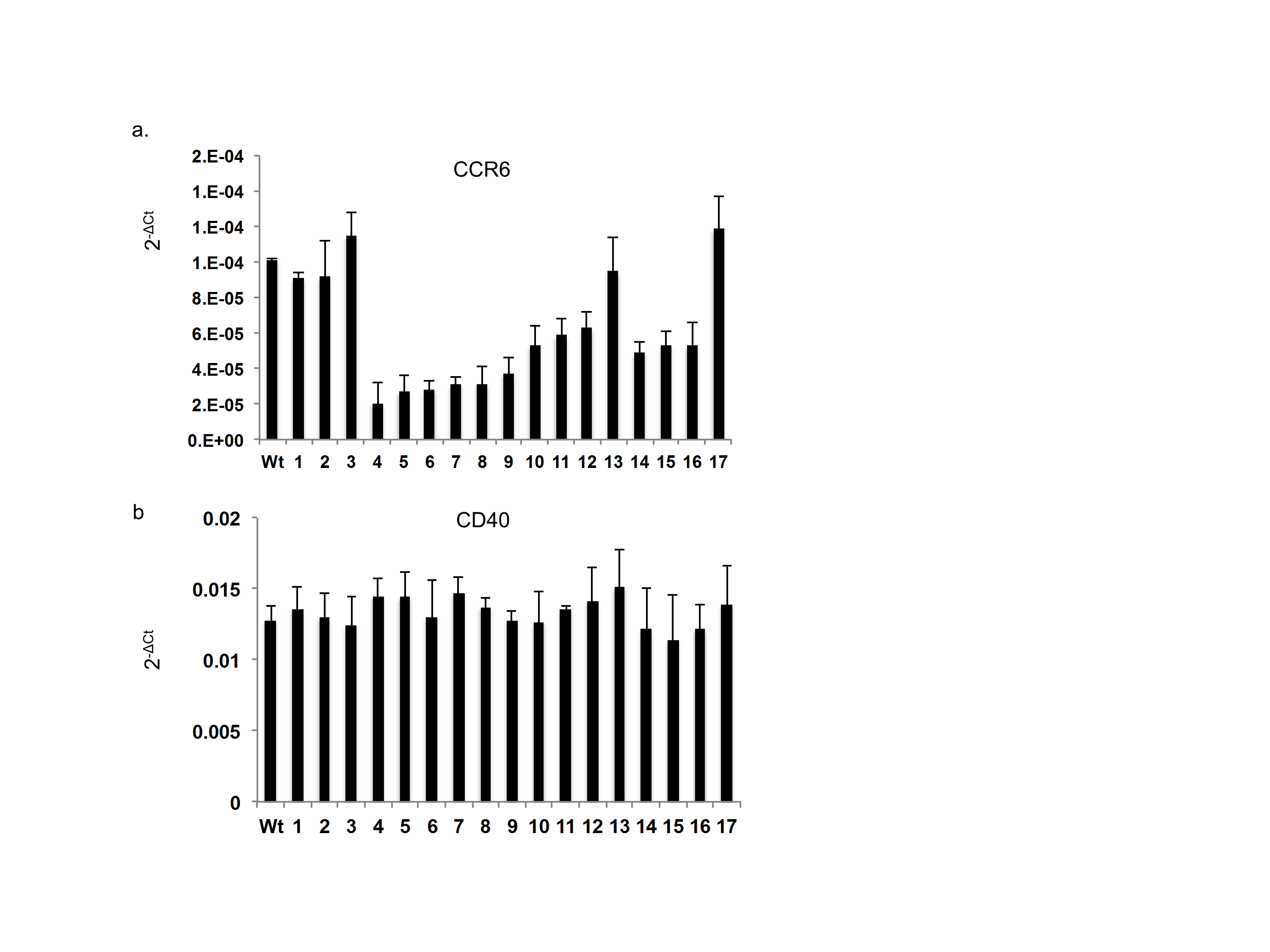

Supplement: S2 Fig — (TIF) [file pgen.1006292.s002.tif]

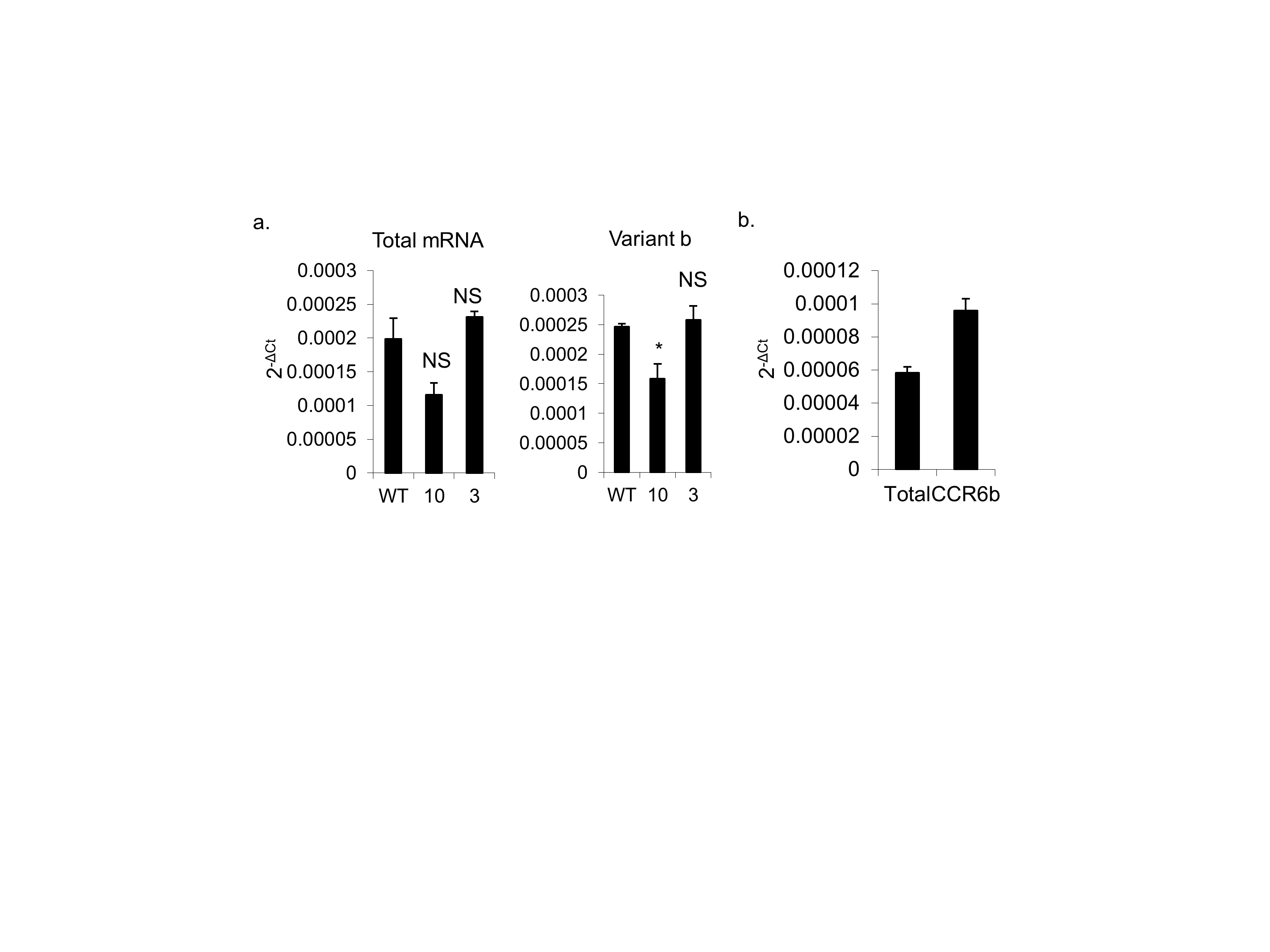

Supplement: S3 Fig — (TIF) [file pgen.1006292.s003.tif]
